# Spindle proteins are differentially expressed in the various histological subtypes of testicular germ cell tumors

**DOI:** 10.4103/1477-3163.60358

**Published:** 2010-03-04

**Authors:** Espen Burum-Auensen, Rolf I. Skotheim, Aasa R. Schjølberg, Jo Røislien, Ragnhild A. Lothe, Ole Petter F. Clausen

**Affiliations:** Division of Pathology, Medical Faculty, University of Oslo, Rikshospitalet, Oslo University Hospital, Oslo, Norway; 1 Department of Cancer Prevention, Institute for Cancer Research, Norwegian Radium Hospital, Oslo University Hospital, Oslo, Norway; 2 Department of Center for Cancer Biomedicine, University of Oslo, Oslo, Norway; 3 Department of Biostatistics, University of Oslo, Oslo, Norway

**Keywords:** Aurora kinases, spindle checkpoint, testicular cancer

## Abstract

**Background:**

Testicular germ cell tumors (TGCTs) are characterized by an aneuploid DNA content. Aberrant expression of spindle proteins such as the Aurora kinases and the spindle checkpoint proteins MAD2 and BUB1B, are thought to contribute to the development of chromosomal instability and DNA aneuploidy in cancer. The importance of these spindle proteins remains unknown in the development of TGCTs, thus we have explored the expression levels of these proteins in normal and malignant testicular tissues.

**Materials and Methods:**

Using tissue microarrays the expression levels of Aurora kinase A (AURKA), Aurora kinase B (AURKB), BUB1B and MAD2 were measured in normal, preneoplastic and malignant testicular tissues of different histological subtypes from 279 orchidectomy specimens by means of immunohistochemistry.

**Results:**

All the spindle proteins except for AURKB were expressed in normal testis. Sixty-eight and 36%, respectively, of the primary spermatocytes in the normal testis were positive for BUB1B and MAD2, while only 5% of the cells were positive for AURKA. There was a significantly lower expression of the spindle checkpoint proteins in carcinoma in situ compared to normal testis (*P*=0.008 and *P*=0.043 for BUB1B and MAD2, respectively), while the level of AURKA was increased, however, not significantly (*P*=0.18). The extent of spindle protein expression varied significantly within the different histological subtypes of TGCTs (*P*<0.001 for AURKB, BUB1B and MAD2, *P*=0.003 for AURKA). The expression of AURKA was significantly elevated in both non-seminomas (*P*=0.003) and seminomas (*P*=0.015). The level of BUB1B was significantly decreased in non-seminomas (*P*<0.001). A similar tendency was observed for MAD2 (*P*=0.11).

**Conclusions:**

In carcinoma in situ of TGCTs the spindle checkpoint proteins MAD2 and BUB1B are significantly less expressed compared to normal testis, while the expression of AURKA is increased. We suggest that these changes may be of importance in the transition from in situ to invasive testicular cancer.

## BACKGROUND

Testicular germ cell tumors (TGCTs) are the most frequent solid malignant tumors diagnosed in men aged 20-40 years, and account for up to 60% of all malignancies diagnosed in this age group.[1] All TGCTs develop from a common preinvasive stage of intratubular germ cell neoplasia (IGCN; i.e. carcinoma *in situ*). IGCN may either retain pluripotency and develop into non-seminomas, or mature along the germinal lineage and develop into seminomas.[2] The TGCT genome possesses a high degree of chromosomal instability and shows extensive DNA aneuploidy.[3] Seminoma is the most common histological subtype, representing 50% of all TGCTs.[4] Generally, seminomas are aneuploid with hypertriploid DNA content showing similar chromosomal changes to IGCN cells.[5] The development of DNA aneuploidy is considered to be one of the earliest changes in cancer development.[6,7]

**Figure 1:**
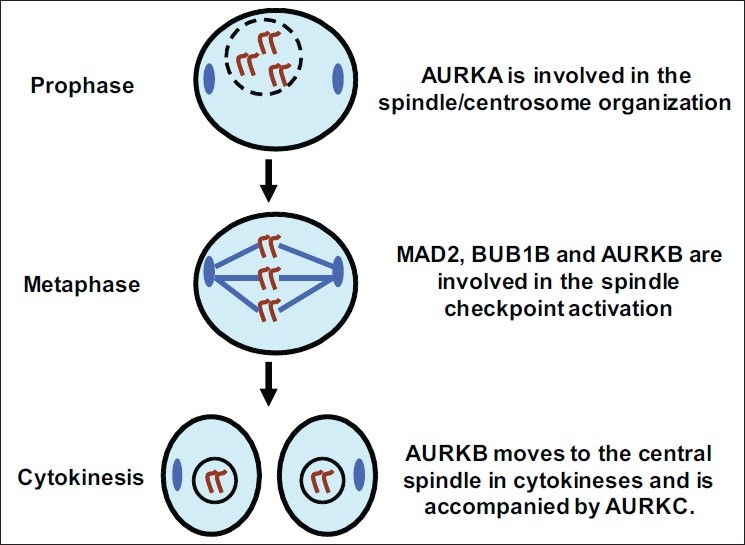
Simplified overview of mitosis with the prophase, metaphase and cytokinesis illustrating the function of the Aurora kinases and the spindle checkpoint proteins MAD2 and BUB1B. In prophase AURKA is localized at the centrosomes (blue color), and its main function involves the maturation and organization of duplicated centrosomes.[46] AURKB is localized to the chromosomal kinetochores (blue lines) during prophase and metaphase,[9] thereafter at the midbody (blue color) during cytokinesis.[12] Detecting the lack of tension at the kinetochores is crucial for spindle checkpoint activation,[11] leading to BUB1B and MAD2-mediated arrest in mitosis until all chromosomes are aligned and bound to a respective microtubule.[24] AURKC is regarded as a chromosomal passenger protein closely related to AURKB,[15] probably cooperating with AURKB in regulating chromosome segregation and cytokinesis

A role for the centrosomes in the early process of aneuploidy development has been shown by Pihan *et al*., reporting centrosome defects occurring in carcinoma *in situ* of prostate, breast and uterine cervical cancers.[8] The mitotic kinase Aurora kinase A (AURKA) is known to be localized to the centrosome[9] and is reported to induce centrosome abnormalities and aneuploidy in human cell lines.[10] Aurora kinase B (AURKB) is also thought to be involved in the development of chromosomal instability. Under normal conditions it binds to the kinetochores during prometaphase and activates the spindle checkpoint upon errors of spindle-kinetochore attachments.[11] During cytokinesis AURKB moves to the contractile ring on the midbody[12] and errors at this point lead to polyploidization.[13] Less is known about the function of the third member of the Aurora kinases, Aurora kinase C (AURKC).[14] AURKC is regarded as a chromosomal passenger protein closely related to AURKB, probably cooperating with AURKB in regulating chromosome segregation and cytokinesis.[15] Figure 1 summarizes the function of these spindle proteins in mitosis.

The mitotic checkpoint consists of several evolutionary conserved proteins including BUB1, BUB1B (i.e. BUBR1), BUB3, MAD1 and MAD2.[16] When chromosomes are not properly attached to the mitotic spindle in mitosis, the hceckpoint inhibits further mitotic progression.[17] BUB1B and MAD2 are two vital components of the mitotic checkpoint that have received attention for their putative roles during the development of aneuploidy and tumorigenesis. In normal human fibroblasts the inhibition of BUB1 leads to genomic instability and anchorage-independent growth.[18] Furthermore, mutant mice with low levels of Bub1b (ortholog to human BUB1B) develop progressive aneuploidy, impaired wound healing, defects in meiotic chromosome segregation and infertility.[19] In nasopharyngeal carcinomas, reduced MAD2 levels have been reported to contribute to chromosomal instability.[20] Based upon these data we wanted to explore the protein expression patterns of the spindle proteins AURKA, AURKB, AURKC, MAD2 and BUB1B in the different histological subtypes of TGCTs, all of which are characterized by aneuploid DNA content which is thought to be essential for cancer development.

## MATERIALS AND METHODS

### Histopathologic evaluation of tumors

The expression of the spindle proteins was examined in non-neoplastic testicular tissue and tumors in a cohort of orchidectomy specimens by using tissue microarrays (TMA). TMAs have previously been used in the investigation of other biological markers during TGCT progression, and clinical data, method for core sampling as well as a description of the histological classification of TGCT is described in this report.[21] In the TMA, samples from 279 individuals are represented by 510 testicular tissue cores. Tissue cores that did not contain representative tissue or showed an insufficient number of tumor cells (< 50 cells), were excluded in the current study, that reduced the number of tissue cores to 357. From 48 of the orchidectomy specimens, tissue cores with more than one morphological differentiation were harvested into the TMA. Of the 357 tissue cores, 21 were from morphologically normal testicular tissue, 17 from IGCN, 135 from seminomas, 71 from embryonal carcinomas, 54 from yolk sac tumors, and 59 were from teratomas. Choriocarcinomas were not analyzed because of insufficient quality of the tissue available.

### Western blot analysis

**Figure 2:**
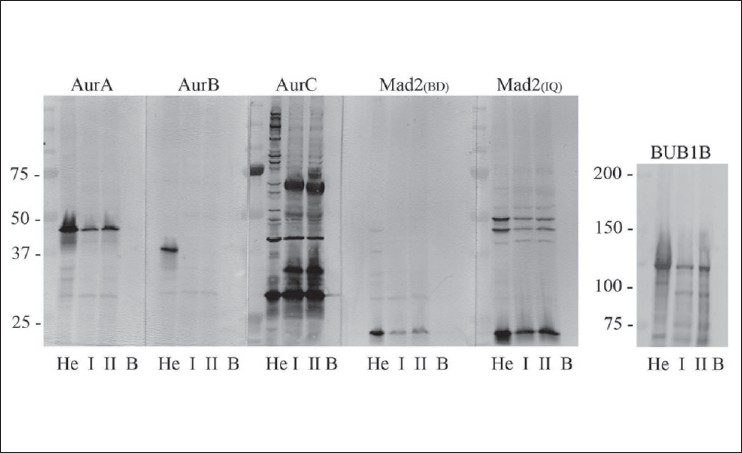
Illustrates the Western blot analyses of the antibodies used and tested against the spindle proteins Aurora A (AURKA, Mw 46 kDa), Aurora B (AURKB, Mw 41 kDa), Aurora C (AURKC, Mw 32 kDa), MAD2 (Mw 24 kDa) and BUB1B (Mw 125 kDa). He; Hela cells, I/II; two suspensions from normal testicular tissues, each a mixture of three different testes. B: Blank. As can be seen, AURKB was not expressed at a detectable level in normal human testicular tissues. The antibody against AURKC showed affinity to several nonspecific antigens, and was omitted from further analyses

Western blot analyses were performed to evaluate the antibodies included [Figure 2]. Samples for blotting were taken from freshly frozen testicular tissue with normal morphology. Due to a limited amount of normal testicular tissue available, biopsies from three patients were mixed to make one suspension, and two suspensions from biopsies of six patients were made. A suspension from a HeLa cell line was included as an unambiguous positive control. The tissues were cut into small pieces, and cells and tissues were boiled in 300 *μ*l Laemmli buffer containing 5 % β mercaptoethanol and 0.5% PMSF (phenylmethylsulphonylfluoride). Protein concentrations were measured (RC DC Protein Assay, BioRad) and 20 *μ*g of protein sample and 5 *μ*g of a molecular weight standard (Precision Plus Protein Standards, BioRad, Hercules CA) were loaded onto SDS-polyacrylamide gel. After electrophoresis samples were transferred onto PVDF (polyvinylidene difluoride) membranes and incubated overnight at 4°C with 1:500 dilution of all primary antibodies of interest (AURKA, 46 kDa, Novocastra, NCL-L-AK2, Newcastle, UK; AURKB, 41 kDa, BD Transduction Laboratories, antibody ID 611083, Franklin Lakes, NY, US; BUB1B, BD Transduction Laboratories, antibody ID 612503). MAD2 was examined with two different antibodies, one from BD Transduction Laboratories (ID 610679), and another from Immuquest (IQ239, Cleveland, UK). Two antibodies against AURKC were examined; one from Zymed Laboratories Inc., diluted 1:125 and the other from Abgent #AP7000g. The antibody from Abgent showed possible cross-reaction to AURKB (data not shown) and was excluded for further analysis. One lane was left empty as a negative methodological control, labeled B for blank [Figure 2]. Blots were then incubated with AP-labeled polymer conjugated secondary antibody for 1 h (Envision+, DakoCytomation, Via Real Carpintera), and developed using a NBT/BCI colorimetric procedure (Roche Diagnostics, Mannheim, Germany).

### Immunohistochemistry

Immunohistochemical analysis of expression of spindle proteins in human tissues has been extensively described by us previously.[22] TMA sections of 4 *μ*m thickness were exposed to a 0.5% H_2_0_2_ solution, then subjected to Tris-EDTA pH 9.0 antigen retrieval buffer for 20 min in a microwave oven (for AURKB retrieval with EDTA at pH 8.0 was used) followed by incubation for 1 h at room temperature with the primary antibodies (AURKA; 1:50 dilution, AURKB; 1:500 dilution, BUB1B; 1:300 dilution). MAD2 was examined with two different antibodies, one from BD Transduction Laboratories ID diluted 1:50, and the other from Immuquest diluted 1:200. The slides were rinsed for 10 min in IHC wash solution (Ventana Medical Systems Inc.), followed by 30 min of incubation with HRP-labeled polymer conjugated secondary antibody (EnVision, DakoCytomation, Via Real Carpintera). DAB+ (DakoCytomation) was applied for seven min, followed by washing in distilled water for 10 min, counterstained with hematoxylin and mounted. Normal tonsils fixed in formalin were used as positive controls, for negative control TBS was substituted for the primary antibody. An average of 350 randomly selected malignant cells were counted in each tissue core, and the cells were evaluated as either positive or negative. The number of positive cancer cells among the 350 evaluated cells was used to calculate the percentage of positive cancer cells in each tissue core. This defines protein expression in this report. However, in the non-seminoma group of cancers the number of tumor cells in the tissues are known to be due to a larger amount of supporting tissue, which reduced the number of cells available for scoring in some cases down to an average of 150 cells per tissue core.

### Antibody purification

Highly confluent HCT116 colon cancer cells expected to express AURKC at very low levels were trypsinized, rinsed in PBS and centrifuged at 1500 X g for 6 min and the supernatant discarded. The pellet was washed twice in PBS and resuspended in 10 ml PBS. One ml of this solution was treated with three cycles of 10-sec sonication on ice. The cell lysate was mixed with the polyclonal antibody against AURKC (Zymed Inc.) to achieve relevant antibody concentration for Western blotting (1:250 for the purification study). After incubation overnight at 4°C, the sample was centrifuged at 2000 X g for 6 min, and the supernatant solution containing the purified antibody was applied for Western blot analysis.

### Statistical analysis

Protein expression was regarded as a continuous variable, thus cutoff values were not implemented. To compare protein expression between the different histological subtypes of TGCTs, both non-parametric methods (Mann-Whitney (MW) and Kruskal-Wallis (KW)) and parametric regression analyses (General linear model (GLM) and the General linear mixed model (GLMM)) were performed. A *P* value of ≤ 0.05 was considered statistically significant. GLM automatically adjusts for biased *P* values based upon multiple statistical analyses, excluding the need for Bonferroni corrections.

Due to the fact that several tissue cores with different cancer morphology were harvested from the same orchidectomy specimens, one might expect that this could bias the statistical analysis as the observations, biopsy samples, are not independent. We therefore reanalyzed the data using the more sophisticated statistical regression tool GLMM. GLMM can be viewed as a combination and extension of different types of ANOVA and regression analyses into the same mathematical framework, where also the fact that individuals contribute with several observations, *i.e.* biopsy samples, can be adjusted for.

The study protocol was in accordance with the Helsinki Declaration and approved by the regional ethical committee for scientific studies on human tissues.

## RESULTS

### Western blot analysis

All antibodies were initially evaluated by Western blot analysis, and all gave specific bands at the expected molecular weights of the corresponding proteins [Figure 2]. The AURKC antibody from Abgent (#AP7000g) showed possible cross-reaction with AURKB on initial immunohistochemical analysis, and the AURKC antibody from Zymed Inc. produced numerous nonspecific bands on Western blot analysis (data not shown). Thus both antibodies against AURKC were excluded from further analyses. The antibody against MAD2 from BD Transduction Laboratories showed the best antigen specificity and was chosen for further analyses.

### Immunohistochemical analysis of normal testis

**Figure 3:**
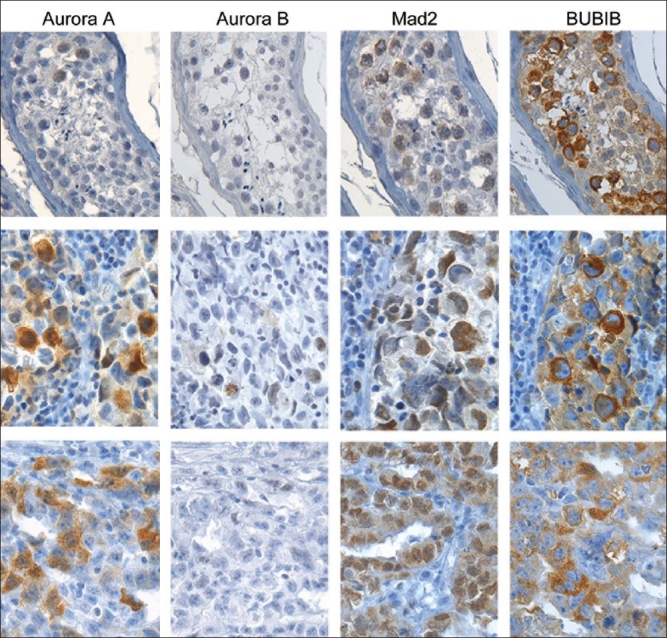
Immunohistochemical analysis of the spindle proteins AURKA, AURKB, MAD2 and BUB1B in normal human testes (upper row), seminomas (middle row) and embryonal carcinomas (lower row). A few spermatocytes weakly positive for AURKA can be seen in normal testis. In the cancerous tissues AURKA was expressed more abundantly. AURKB was not expressed in normal testis, and the extent of AURKB expression in cancerous tissues was very limited. The spindle checkpoint proteins MAD2 and BUB1B were highly expressed in both normal and cancerous tissues

**Figure 4:**
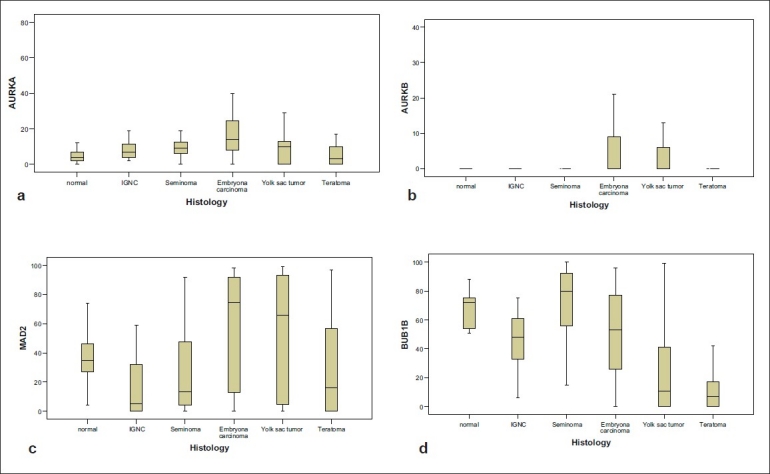
Protein expression of AURKA (a), AURKB (b), MAD2 (c) and BUB1B (d) in the different histological subtypes of TGCT

The results from the immunohistochemical analysis are shown in Table 1, Figures 3 and 4. In the normal testis, none of the antibodies gave any immunoreactivity in Leydig cells, Sertoli cells or spermatids. AURKA was localized to the nucleus of 5% primary spermatocytes, with accompanying weak staining of the cytoplasm in some cells [Figure 3]. Some spermatogonia were also positive for AURKA. MAD2 showed similar staining patterns as AURKA, with positive nuclei in 36% of spermatocytes with accompanying weak staining of the cytoplasm in some cells. Spermatogonia were rarely positive for MAD2. BUB1B showed strong cytoplasmic staining of 68% of primary spermatocytes and some spermatogonia although the staining intensity was lower in the latter cell type. Cells positive for AURKB were not detected in normal testis. All proteins were expressed in the germinal centers of tonsils that were included as positive controls. Sections in which TBS was substituted for the primary antibody were negative.

**Table 1:** Summary of spindle protein expression (in percent) in the different histological subtypes of TGCTs and results from the statistical analyses (KW; Kruskal-Wallis, GLM; general linear model, MW; Mann-Whitney)

Histological subtypes	AURKA	AURKB	BUB1B	MAD2
Normal testis	5	0	68	36
IGCN	8	<1	46	15
Seminoma	10	1	73	27
Embryonal carcinoma	16	6	51	58
Yolk sac tumor	9	3	25	52
Teratoma	5	1	16	30
All histological subtypes (KW)		*P*<0.001	*P*<0.001	*P*<0.001
Normal vs. IGCN (GLM)		*P*=0.18	*P*=0.08	*P*=0.043
Seminoma vs. normal (GLM)		*P*=0.015	*P*=0.70	*P*=0.17
Nonseminoma vs. normal (GLM)		*P*=0.003	*P*<0.001	*P*=0.11
Seminoma vs. nonseminoma (MW)		*P*=0.3	*P*<0.001	*P*=0.001

**Supplementary Table 1:** Number of representative tissue cores used for the immunohistochemical analysis of the different antibodies

	Frequency	Percent	AURKA	AURKB	BUB1B	MAD2
Normal testis	21	5.9	21	21	21	21
IGCN	17	4.8	16	17	17	17
Seminoma	135	37.8	131	133	131	132
Embryonal Carcinoma	71	19.9	63	65	69	68
Yolk sac tumor	54	15.1	49	51	50	48
Teratoma	59	16.5	54	54	49	43
Total	357	100	334	341	337	329

### Immunohistochemical analysis of the histological subtypes of TGCTs

Although not statistically significant, the level of AURKA was higher in IGCN compared to normal testis (*P*=0.18). The level of AURKA differed significantly within the different histological subtypes of TGCTs (*P*<0.001), and was significantly higher in both seminomas and non-seminomas compared to normal testis (*P*=0.015 and *P*=0.003, respectively). The level of AURKA was highest in embryonal carcinomas (16%), whereas teratomas showed the same level as normal testis (5%). Although AURKB could not be detected in normal testis, it was expressed at very low levels in the different histological subtypes of TGCTs [Table 1]. Due to the very low levels of expression, AURKB was excluded from the statistical analysis.

The levels of spindle checkpoint proteins were generally decreased in IGCN compared to normal testis (*P*=0.008 and *P*=0.043 for BUB1B and MAD2, respectively). As for AURKA, the differences in protein expression levels of BUB1B in the different histological subtypes of TGCTs were highly significant (*P*<0.001). BUB1B expression was significantly reduced in non-seminomas compared to normal testis (*P*<0.001). In seminomas, BUB1B expression was similar to that of normal testis, and significantly higher compared to the non-seminomas (*P*<0.001). MAD2 expression, however, showed an opposite expression pattern with higher levels in the non-seminoma group compared to seminomas (*P*<0.001). Within the different histological subtypes of TGCTs, the differences in MAD2 expression were highly significant (*P*<0.001). In contrast to the other spindle proteins, MAD2 expression in seminomas and non-seminomas was not significantly different from that in normal testis (*P*=0.17 and *P*=0.11 for seminoma and nonseminoma, respectively). Reanalyzing the data using GLMM did not change any of the significant results obtained by the nonparametric analyses.

## DISCUSSION

To the authors' knowledge, this is the first immunohistochemical study of BUB1B in TGCTs. In normal testis the level of BUB1B reached nearly 70% [Figure 3, Table 1], which is in contrast to the low levels reported in other human tissues.[23] BUB1B as well as MAD2 are key spindle checkpoint proteins, critical for normal cell division and tissue development. Spermatogenesis is a unique developmental process where diploid stem cells differentiate into haploid spermatozoa, including both mitotic and meiotic cell divisions. Occasionally, chromosomes fail to separate normally during meiosis, a phenomenon called nondisjunction. The frequency of such erroneous segregations during meiosis in the female germ cells is remarkably high and about 10%, which may be one reason for the high rate of miscarriages in early pregnancy. During normal cell division, the lack of tension across the kinetochore in cases of nondisjunction is detected by BUB1B,[24] which activates the spindle checkpoint and halts the mitosis until the spindle apparatus and chromatids are aligned correctly. The high levels of BUB1B observed in normal testis might be explained by the importance of avoiding any errors during these critical cell divisions. The consequences of such errors for the organism to develop are dramatic. This is seen in families with missense mutations of BUB1B, which results in Mosaic Variegated Aneuploidy (MVA), a syndrome characterized by aneuploidy, growth retardation and childhood cancer.[25,26]

There are several lines of evidence that IGCN develop from embryonal germ cells during the fetal life.[2] Such material has not been available for analysis in this study, thus we cannot conclude about the levels of spindle proteins at this putative early stage of tumorigenesis, but we have included analyses of normal testicular tissues. Both BUB1B and MAD2 expression were reduced in IGCN as compared to normal testis, which is consistent with the suggestion that downregulation of the spindle checkpoint proteins might be an early preneoplastic event.[27] The level of BUB1B expression was reduced in all the different histological subtypes of TGCTs when compared to normal testis, with the exception of seminomas. This is probably explained by the fact that seminomas have mainly retained their phenotypic characteristics of the spermatocytes, whereas the other TGCTs display a variety of phenotypes. We have recently shown that BUB1B is overexpressed in colorectal cancer compared to normal colonic mucosa,[28] also consistent with results from bladder cancer.[29] However, in contrast to testicular tissue, BUB1B levels are low in most other normal human tissues, consistent with their low mitotic activities. Normal testis represents a unique tissue in this respect, since there is a considerable accumulation of tetraploid spermatocytes in the G2 phase of the cell cycle due to the long duration of this cell cycle phase in the testis. BUB1B is closely associated with the process of cell division and is known to be expressed in G2 cells,[23] which explains the high level observed in the testis.

The decreased level of MAD2 in IGCN compared to normal testis is in agreement with another study of MAD2 expression in TGCT, suggesting that downregulation of MAD2 plays a role in an impaired spindle checkpoint and CIN observed in TGCT.[30] This study also reports of less nuclear and relatively increased cytoplasmatic MAD2 levels as an explanation for an impaired spindle checkpoint function in TGCT. Our immunohistochemical analysis is consistent with that observation [Figure 3]. Another report supporting these findings is the *in vitro* study by Kasai and collaborators[31] on MAD2 in human T-cell leukemia virus Type I transformed cells. This report revealed that the dislocation of MAD2 from the nucleus to the cytoplasm was correlated with loss of spindle checkpoint function in these cells. Such a loss of function may be related to that MAD2 localized in the cytoplasm displaced from its binding motifs in the nucleus, prevents it to fulfill its role as a spindle checkpoint mediator. However, these results remain to be confirmed *in vivo*. The level of MAD2 in the non-seminomas was increased compared to normal controls, in contrast to what was seen in the seminoma group. The difference in MAD2 levels between these two histological subtypes was highly significant, suggesting that the spindle checkpoint is not downregulated in non-seminomas. Similar to the expression of Aurora kinases, both the spindle checkpoint proteins were expressed at the lowest levels in teratomas, which may be explained by the retained spermatocytic differentiation of this tissue having a low proliferative index.

The increased expression of AURKA in testicular neoplasms is in agreement with other studies of this kinase in malignant human tumors.[32–35] The level of AURKA expression in IGCN was also increased, although not significantly when compared to normal testis, consistent with observations from *in situ* carcinomas of the breast[36] and in ovarian carcinoma.[37] AURKA overexpression has a critical role in the development of DNA aneuploidy by inducing centrosome amplification[10] which is observed in *in situ* carcinomas of the prostate, breast and uterine cervical cancers.[8] Mayer *et al.*,[38] showed that centrosome amplification was associated with aneuploidy in TGCT. They also reported that the centrosome amplification was independent of the AURKA levels. One should interpret these data with some caution, however, based on the low number of TGCTs (n=17) included in this study. Anand and collaborators[39] have demonstrated that overexpression of AURKA overrides an activated spindle checkpoint. Taken together, the downregulation of the spindle checkpoint proteins MAD2 and BUB1B, combined with the increased levels of AURKA as shown in IGCN in this report, suggest that a dysregulation of spindle proteins may be involved in the steps of malignant transformation in testis.

AURKB expression was not detected in normal testis, and only very low levels were detected in the different histological subtypes of TGCTs. This is in contrast to data from Chieffi and collaborators[40] which report AURKB expression in both normal and malignant testicular tissues. Our immunohistochemical results are supported by Western blot analysis of normal, human testicular tissue where AURKB was not detected [Figure 1]. The Western blot analyses by Chieffi *et al.*, were performed on lysates from mouse testis, and demonstrate that murine spermatozoa, spermatids and spermatocytes actually do not express AURKB. The only cells positive for AURKB were spermatogonia. In other tissues such as the thyroid gland,[41] brain[42] and colon[28] AURKB was found to be overexpressed in cancers compared to normal tissues. It has been shown that increased levels of AURKB cause genetic instability and cancer due to spindle checkpoint anomalies and polyploidization,[13] however, the low levels of AURKB detected in this report indicate a minor role for this kinase in TGCT.

*In vitro* studies of MAD2 show that downregulation in TGCT cell lines is associated with decreased sensitivity to cisplatin[43] and similar results are reported from both nasopharyngeal[44] and ovarian cancer cell lines. Similarly, cells devoid of BUB1B expression fail to stop at checkpoints after DNA damage induced by irradiation and after doxorubicin treatment.[45] These *in vitro* studies indicate that the spindle checkpoint proteins might serve as markers of prognosis as well as predictors of appropriate treatment strategies.[46]

## CONCLUSIONS

The spindle checkpoint proteins MAD2 and BUB1B are expressed at low levels in the preneoplastic stages of TGCTs. The level of these spindle proteins varies significantly between the different histological subtypes of TGCTs. AURKA expression is increased in both IGCN and TGCTs, and downregulation of the spindle checkpoint proteins together with elevated levels of AURKA in IGCN may be of importance in the transition from *in situ* to invasive testicular cancer.

## COMPETING INTERESTS

The authors declare that they have no competing interests.

## AUTHORS' CONTRIBUTIONS

Ragnhild A. Lothe and Rolf I. Skotheim collected and constructed the TMAs. Espen Burum-Auensen and Aasa R Schjølberg carried out the immunohistochemical and Western blot analyses. Espen Burum-Auensen and Rolf I. Skotheim drafted the article. Jo Røislien performed the statistical analysis. Ragnhild A. Lothe, Ole Petter F. Clausen, Aasa R Schjølberg and Jo Røislien revised the paper critically. All authors contributed substantially to the conception and design of the study, and all authors have given a final approval of the version submitted.

## AUTHOR'S PROFILE

**Dr. Jo Roeslien Jr.** Department of Biostatistics, University of Oslo, Oslo, Norway.

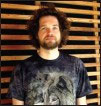



**Dr. Espen Burum-Auensen Jr.** 2007- ENT resident, Akershus University Hospital, Norway 2004-2007 PhD student, Dpt. of Pathology. University of Oslo, Rikshospitalet, Norway 2002-2004 Junior doctor, Kongsvinger regional Hospital. 1995-2001 Semmelweis University of Medicine, Budapest, Hungary.

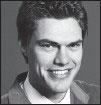



**Prof. Rolf I. Skotheim** Department of Cancer Prevention, Institute for Cancer Research, Norwegian Radium Hospital, Oslo University Hospital, Oslo, Norway

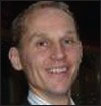



**Prof. Ole Petter Fraas Clausen Sr.** The Pathology Clinic, Institute of Pathology, Rikshospitalet, Oslo University Hospital, Oslo, Norway

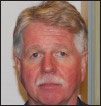



**Prof. Ragnhild Lothe Sr.** Department of Cancer Prevention, Institute for Cancer Research, Norwegian Radium Hospital, Oslo University Hospital, Oslo, Norway

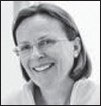



**Mrs. Aasa R. Schjoelberg Sr.** Department of Cancer Prevention, Institute for Cancer Research, Norwegian Radium Hospital, Oslo University Hospital, Oslo, Norway


